# Gene model for the ortholog of *dock* in *Drosophila eugracilis*

**DOI:** 10.17912/micropub.biology.001848

**Published:** 2025-09-11

**Authors:** Gabriella N. Bicanovsky, Megan E. Lawson, Alexandria Groeneveld, Jordan Elkinton, Eden Dowell, S. F. Shelley-Tremblay, Stephanie Toering Peters, Lindsey J. Long, Anya Goodman, Melinda A. Yang, Chinmay P. Rele, Laura K. Reed

**Affiliations:** 1 The University of Alabama, Tuscaloosa, AL USA; 2 Wartburg College, Waverly, IA, USA; 3 Oklahoma Christian University, Edmond, OK USA; 4 Wartburg College, Waverly, IA USA; 5 California Polytechnic State University, San Luis Obispo, CA USA; 6 University of Richmond, Richmond, VA USA

## Abstract

Gene model for the ortholog of dreadlocks ( *dock* ) in the *D. eugracilis* Apr. 2013 (BCM-HGSC/Deug_2.0) (DeugGB2) Genome Assembly (GenBank Accession: GCA_000236325.2 ) of *Drosophila eugracilis* . This ortholog was characterized as part of a developing dataset to study the evolution of the Insulin/insulin-like growth factor signaling pathway (IIS) across the genus *Drosophila* using the Genomics Education Partnership gene annotation protocol for Course-based Undergraduate Research Experiences.

**Figure 1. Genomic neighborhood and gene model for dock in Drosophila eugracilis:**
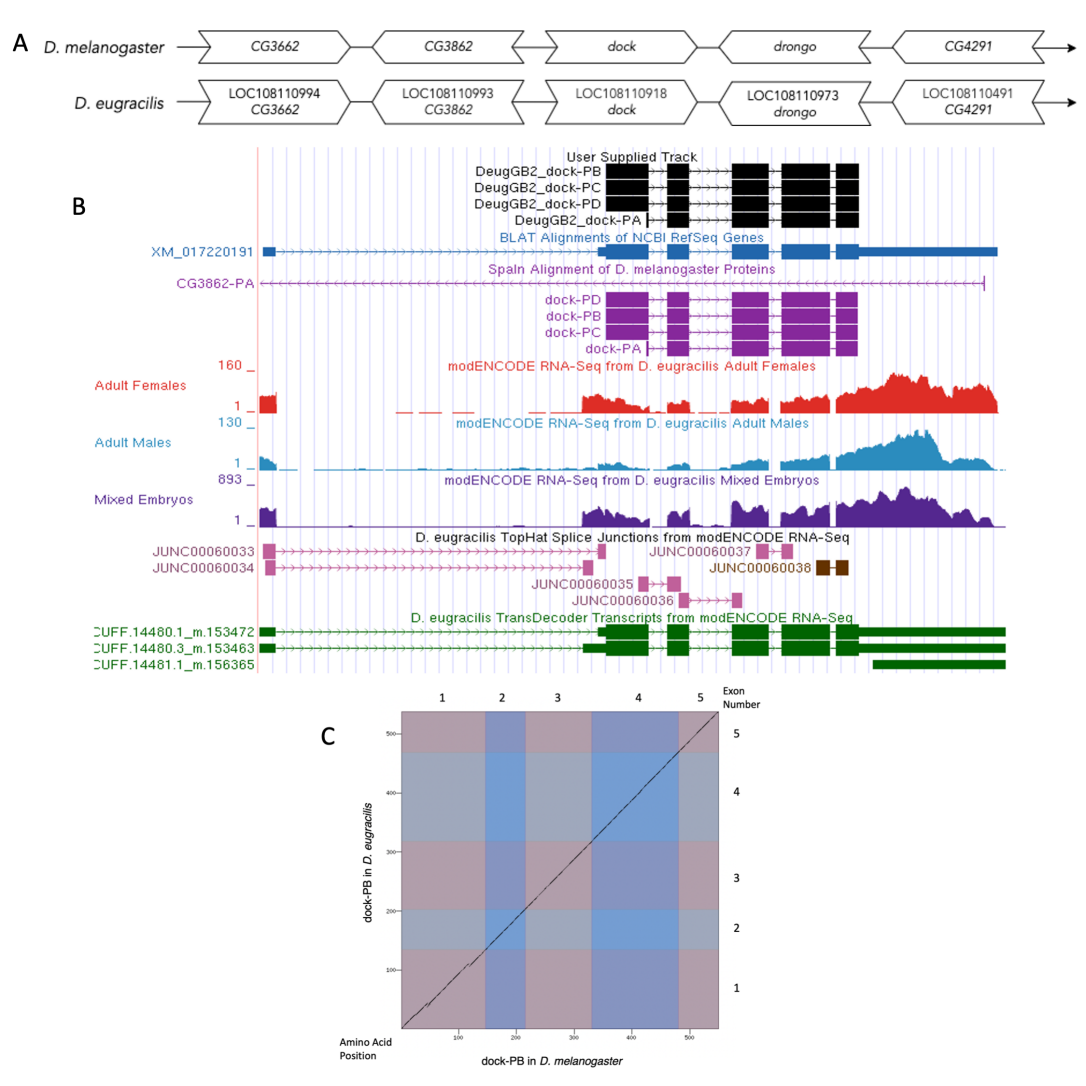
**(A) Synteny** **of genomic neighborhood of *dock* in *D. melanogaster* and *D. eugracilis* .** Gene arrows pointing in the same direction as target gene in both *D. eugracilis* and *D. melanogaster* are on the same strand as the target gene; gene arrows pointing in the opposite direction are on the opposite strand. The thin underlying arrows pointing to the right indicate that *dock* is on the + strand. White arrows in *D. eugracilis* indicate the locus ID and the orthology to the corresponding gene in *D. melanogaster* . The gene names given in the *D. eugracilis* gene arrows indicate the orthologous gene in *D. melanogaster* , while the locus identifiers are specific to *D. eugracilis* . **(B)** **Gene Model in UCSC Track Hub** (Raney et al., 2014): the gene model in *D. eugracilis* (black), Spaln of D. melanogaster Proteins (purple, alignment of Ref-Seq proteins from *D. melanogaster* ), BLAT alignments of NCBI Ref-Seq Genes (blue, alignment of Ref-Seq genes for *D. eugracilis* ), RNA-Seq from adult females (red), adult males (blue), and mixed embryos (purple), alignment of Illumina RNA-Seq reads from *D. eugracilis* , and Transcripts (green) including coding regions predicted by TransDecoder and Splice Junctions Predicted by regtools using *D. eugracilis* RNA-Seq (PRJNA63469). Splice junctions shown have a minimum read-depth of 105 with 100-499 and 500-999 supporting reads in pink and brown, respectively. The custom gene model (User Supplied Track) is indicated in black with exon depicted with wide boxes, intron with narrow lines (arrows indicate direction of transcription). **(C) Dot Plot of dock-PB in *D. melanogaster* ( *x* -axis) vs. the orthologous peptide in *D. eugracilis* ( *y* -axis)** . Amino acid number is indicated along the left and bottom; coding exon (CDS) number is indicated along the top and right, and CDSs are also highlighted with alternating colors.

## Description

**Table:** 

* This article reports a predicted gene model generated by undergraduate work using a structured gene model annotation protocol defined by the Genomics Education Partnership (GEP; thegep.org ) for Course-based Undergraduate Research Experience (CURE). The following information in this box may be repeated in other articles submitted by participants using the same GEP CURE protocol for annotating Drosophila species orthologs of Drosophila melanogaster genes in the insulin signaling pathway. * "In this GEP CURE protocol students use web-based tools to manually annotate genes in non-model *Drosophila* species based on orthology to genes in the well-annotated model organism fruitfly *Drosophila melanogaster* . The GEP uses web-based tools to allow undergraduates to participate in course-based research by generating manual annotations of genes in non-model species (Rele et al., 2023). Computational-based gene predictions in any organism are often improved by careful manual annotation and curation, allowing for more accurate analyses of gene and genome evolution (Mudge and Harrow 2016; Tello-Ruiz et al., 2019). These models of orthologous genes across species, such as the one presented here, then provide a reliable basis for further evolutionary genomic analyses when made available to the scientific community.” (Myers et al., 2024). “The particular gene ortholog described here was characterized as part of a developing dataset to study the evolution of the Insulin/insulin-like growth factor signaling pathway (IIS) across the genus *Drosophila* . The Insulin/insulin-like growth factor signaling pathway (IIS) is a highly conserved signaling pathway in animals and is central to mediating organismal responses to nutrients (Hietakangas and Cohen 2009; Grewal 2009).” (Myers et al., 2024). “ *D. eugracilis* (NCBI:txid29029) is part of the *melanogaste* r species group within the subgenus *Sophophora* of the genus *Drosophila* (Pélandakis et al., 1993). It was first described as *Tanygastrella gracilis* by Duda (1924) and revised to *Drosophila eugracilis* by Bock and Wheeler (1972). *D. eugracilis* is found in humid tropical and subtropical forests across southeast Asia (https://www.taxodros.uzh.ch, accessed 1 Feb 2023).” (Morgan et al., 2022).

We propose a gene model for the *D. eugracilis* ortholog of the *D. melanogaster* *dreadlocks* ( *dock* ) gene. The genomic region of the ortholog corresponds to the uncharacterized protein XP_017075680.1 (Locus ID LOC108110918 ) in the Apr. 2013 (BCM-HGSC.DEUG_2.0) Genome Assembly of *D. eugracilis* ( GCA_000236325.2 ). This model is based on RNA-Seq data from *D. eugracilis* ( PRJNA63469 ; Chen et al., 2014) and *dock* in *D. melanogaster* using FlyBase release FB2023_03 ( GCA_000001215.4 ; Larkin et al., 2021; Gramates et al., 2022; Jenkins et al., 2022).

The product of gene *dreadlocks* ( *dock* , FBgn0010583) is involved in several cellular functions, including axon guidance (Garrity et al., 1996; Hing et al., 1999; Schmucker et al., 2000; Stevens and Jacobs 2002; Weng et al., 2011), myoblast fusion during muscle fiber formation (Kaipa et al., 2013), regulation of intercellular bridges in germline cells during gametogenesis (Stark et al., 2021), and negative regulation of insulin receptor signaling pathway (Wu et al., 2011; Willoughby et al., 2017). A *dock* transcript was first isolated and sequenced in *Drosophila melanogaster* in a screen for P-element insertions leading to R cell projection defects (Garrity et al., 1996). *dock* encodes a protein that contains three N-terminal SH3 domains and one C-terminal SH2 domain known to bind to specific motifs and serve as binding adaptors (Garrity et al., 1996). Its regulation of photoreceptor axon guidance in *Drosophila* occurs through its interaction with InR (Rao et al., 1998; Song et al., 2003; Rao 2005). The dock protein plays a role in negatively regulating the insulin signaling pathway by facilitating the dephosphorylation of InR through recruitment of the ER-localized form of protein tyrosine phosphatase PTP61F, a function that is also observed in its mammalian ortholog *Nck* (Wu et al., 2011; Buszard et al., 2013).

***Synteny***

*dock* occurs on *chr2L* in *D. melanogaster* and is flanked by upstream genes *CG3662* and *CG3862* and downstream genes *drongo* ( *drongo* ) and *CG4291* . It has been determined that the putative ortholog of *dock* is found on scaffold KB465133.1 in *D. eugracilis* (GB2 assembly *GCA_000236325.2* ) with LOC108110918 ( XP_017075680.1 , via *tblastn* search with an e-value of 6e-164 and percent identity of 82.47%), where it is surrounded upstream by LOC108110994 ( XP_017075791.1 ) and LOC108110993 ( XP_017075790.2 ) which correspond to *CG3662* and *CG3862* in *D. melanogaster* with e-values of 0.0, and percent identity of 89.04% and 93.39%, respectively, as determined by *blastp* ( Figure 1A, Altschul et al., 1990). The putative ortholog is flanked with downstream genes LOC108110973 ( XP_017075764.2 ) and LOC108110491 ( XP_017075058.1 ) which correspond to *drongo* and *CG4291* in *D. melanogaster* with e-values of 0.0, and percent identity of 85.61% and 86.98%, respectively, as determined by *blastp* . This is likely the correct ortholog assignment for *dock* in *D. eugracilis* for three reasons: first, a *tblastn* search with *D. melanogaster* dock-PA sequence against the *D. eugracilis* genome generated a top hit at this location with an E-value of 6e-164 and a percent identity of 82.475%. Second, a *blastp* search with the peptide sequence from *D. eugracilis* ( XP_017075680.1 ) against the refseq_protein database in *D. melanogaster* generated a top hit of dock-PD with an e-value of 0.0 and percent identity of 87.43%. Third, the local synteny is highly conserved between the two genomic neighborhoods, with all the surrounding genes being orthologous to each other. The high similarity between the genomic neighborhood and gene identity is expected as *D. eugracilis* is closely related to *D. melanogaster* .

***Protein Model***

*dock* in *D. eugracilis* has encodes two unique protein isoforms; one encoded by mRNAs *dock–RB* , *dock-RC* , and *dock-RD* that all have identical CDSs, while *dock-RA* is unique ( Figure 1B ). All mRNA isoforms contain five CDSs. Relative to the ortholog in *D. melanogaster* , the RNA CDS number and protein isoform count are conserved. When comparing the protein alignment of dock-PB between *D. eugracilis* and *D. melanogaster* there is a high level of protein similarity and an 87.43% amino acid identity as determined by *blastp* ( Figure 1C ). The coordinates of the curated gene models can be found in NCBI at GenBank/BankIt using the accessions BK064454 , BK064455 , BK064456 , and BK064457 . These data are also available in Extended Data files below, which are archived in CaltechData.

## Methods

Detailed methods including algorithms, database versions, and citations for the complete annotation process can be found in Rele et al. (2023). Briefly, students use the GEP instance of the UCSC Genome Browser v.435 ( https://gander.wustl.edu ; Kent WJ et al., 2002; Navarro Gonzalez et al., 2021) to examine the genomic neighborhood of their reference IIS gene in the *D. melanogaster* genome assembly (Aug. 2014; BDGP Release 6 + ISO1 MT/dm6). Students then retrieve the protein sequence for the *D. melanogaster* reference gene for a given isoform and run it using *tblastn* against their target *Drosophila* species genome assembly on the NCBI BLAST server ( https://blast.ncbi.nlm.nih.gov/Blast.cgi ; Altschul et al., 1990) to identify potential orthologs. To validate the potential ortholog, students compare the local genomic neighborhood of their potential ortholog with the genomic neighborhood of their reference gene in *D. melanogaster* . This local synteny analysis includes at minimum the two upstream and downstream genes relative to their putative ortholog. They also explore other sets of genomic evidence using multiple alignment tracks in the Genome Browser, including BLAT alignments of RefSeq Genes, Spaln alignment of *D. melanogaster* proteins, multiple gene prediction tracks (e.g., GeMoMa, Geneid, Augustus), and modENCODE RNA-Seq from the target species. Detailed explanation of how these lines of genomic evidenced are leveraged by students in gene model development are described in Rele et al. (2023). Genomic structure information (e.g., CDSs, intron-exon number and boundaries, number of isoforms) for the *D. melanogaster* reference gene is retrieved through the Gene Record Finder ( https://gander.wustl.edu/~wilson/dmelgenerecord/index.html ; Rele et al *.,* 2023). Approximate splice sites within the target gene are determined using *tblastn* using the CDSs from the *D. melanogaste* r reference gene. Coordinates of CDSs are then refined by examining aligned modENCODE RNA-Seq data, and by applying paradigms of molecular biology such as identifying canonical splice site sequences and ensuring the maintenance of an open reading frame across hypothesized splice sites. Students then confirm the biological validity of their target gene model using the Gene Model Checker ( https://gander.wustl.edu/~wilson/genechecker/index.html ; Rele et al., 2023), which compares the structure and translated sequence from their hypothesized target gene model against the *D. melanogaster* reference gene model. At least two independent models for a gene are generated by students under mentorship of their faculty course instructors. Those models are then reconciled by a third independent researcher mentored by the project leaders to produce the final model. Note: comparison of 5' and 3' UTR sequence information is not included in this GEP CURE protocol (Gruys et al., 2025).

## Data Availability

Description: A GFF, FASTA, and PEP of the model. Resource Type: Model. DOI: https://doi.org/10.22002/b9xm7-ren93

## References

[R1] Altschul SF, Gish W, Miller W, Myers EW, Lipman DJ (1990). Basic local alignment search tool.. J Mol Biol.

[R2] Bock IR, Wheeler MR. 1972. The Drosophila melanogaster species group. Univ. Texas Publs Stud. Genet. 7(7213): 1-102. FBrf0024428

[R3] Buszard BJ, Johnson TK, Meng TC, Burke R, Warr CG, Tiganis T (2013). The nucleus- and endoplasmic reticulum-targeted forms of protein tyrosine phosphatase 61F regulate Drosophila growth, life span, and fecundity.. Mol Cell Biol.

[R4] Chen ZX, Sturgill D, Qu J, Jiang H, Park S, Boley N, Suzuki AM, Fletcher AR, Plachetzki DC, FitzGerald PC, Artieri CG, Atallah J, Barmina O, Brown JB, Blankenburg KP, Clough E, Dasgupta A, Gubbala S, Han Y, Jayaseelan JC, Kalra D, Kim YA, Kovar CL, Lee SL, Li M, Malley JD, Malone JH, Mathew T, Mattiuzzo NR, Munidasa M, Muzny DM, Ongeri F, Perales L, Przytycka TM, Pu LL, Robinson G, Thornton RL, Saada N, Scherer SE, Smith HE, Vinson C, Warner CB, Worley KC, Wu YQ, Zou X, Cherbas P, Kellis M, Eisen MB, Piano F, Kionte K, Fitch DH, Sternberg PW, Cutter AD, Duff MO, Hoskins RA, Graveley BR, Gibbs RA, Bickel PJ, Kopp A, Carninci P, Celniker SE, Oliver B, Richards S (2014). Comparative validation of the D. melanogaster modENCODE transcriptome annotation.. Genome Res.

[R5] Duda, O. 1924. Revision der europäischen u. grönländischen sowie einiger sudostasiat. Arten der Gattung Piophila Fallén (Dipteren) [part]. Konowia 3: 97-113 [1924.07.10]

[R6] Garrity PA, Rao Y, Salecker I, McGlade J, Pawson T, Zipursky SL (1996). Drosophila photoreceptor axon guidance and targeting requires the dreadlocks SH2/SH3 adapter protein.. Cell.

[R7] Gramates LS, Agapite J, Attrill H, Calvi BR, Crosby MA, Dos Santos G, Goodman JL, Goutte-Gattat D, Jenkins VK, Kaufman T, Larkin A, Matthews BB, Millburn G, Strelets VB, the FlyBase Consortium. (2022). Fly Base: a guided tour of highlighted features.. Genetics.

[R8] Grewal SS (2008). Insulin/TOR signaling in growth and homeostasis: a view from the fly world.. Int J Biochem Cell Biol.

[R9] Grewal SS (2008). Insulin/TOR signaling in growth and homeostasis: a view from the fly world.. Int J Biochem Cell Biol.

[R10] Gruys ML, Sharp MA, Lill Z, Xiong C, Hark AT, Youngblom JJ, Rele CP, Reed LK (2025). Gene model for the ortholog of Glys in Drosophila simulans.. MicroPubl Biol.

[R11] Hietakangas V, Cohen SM (2009). Regulation of tissue growth through nutrient sensing.. Annu Rev Genet.

[R12] Hing H, Xiao J, Harden N, Lim L, Zipursky SL (1999). Pak functions downstream of Dock to regulate photoreceptor axon guidance in Drosophila.. Cell.

[R13] Jenkins VK, Larkin A, Thurmond J, FlyBase Consortium (2022). Using FlyBase: A Database of Drosophila Genes and Genetics.. Methods Mol Biol.

[R14] Kaipa BR, Shao H, Schäfer G, Trinkewitz T, Groth V, Liu J, Beck L, Bogdan S, Abmayr SM, Önel SF (2012). Dock mediates Scar- and WASp-dependent actin polymerization through interaction with cell adhesion molecules in founder cells and fusion-competent myoblasts.. J Cell Sci.

[R15] Kent WJ, Sugnet CW, Furey TS, Roskin KM, Pringle TH, Zahler AM, Haussler D (2002). The human genome browser at UCSC.. Genome Res.

[R16] Larkin A, Marygold SJ, Antonazzo G, Attrill H, Dos Santos G, Garapati PV, Goodman JL, Gramates LS, Millburn G, Strelets VB, Tabone CJ, Thurmond J, FlyBase Consortium. (2021). FlyBase: updates to the Drosophila melanogaster knowledge base.. Nucleic Acids Res.

[R17] Morgan A, Kiser CA, Bronson I, Lin H, Guillette N, McMahon R, Kennell JA, Long LJ, Reed LK, Rele CP (2022). Drosophila eugracilis - Akt.. MicroPubl Biol.

[R18] Mudge JM, Harrow J (2016). The state of play in higher eukaryote gene annotation.. Nat Rev Genet.

[R19] Myers A, Hoffman A, Natysin M, Arsham AM, Stamm J, Thompson JS, Rele CP, Reed LK (2024). Gene model for the ortholog Myc in Drosophila ananassae.. MicroPubl Biol.

[R20] Navarro Gonzalez J, Zweig AS, Speir ML, Schmelter D, Rosenbloom KR, Raney BJ, Powell CC, Nassar LR, Maulding ND, Lee CM, Lee BT, Hinrichs AS, Fyfe AC, Fernandes JD, Diekhans M, Clawson H, Casper J, Benet-Pagès A, Barber GP, Haussler D, Kuhn RM, Haeussler M, Kent WJ (2021). The UCSC Genome Browser database: 2021 update.. Nucleic Acids Res.

[R21] Pélandakis M, Solignac M (1993). Molecular phylogeny of Drosophila based on ribosomal RNA sequences.. J Mol Evol.

[R22] Raney BJ, Dreszer TR, Barber GP, Clawson H, Fujita PA, Wang T, Nguyen N, Paten B, Zweig AS, Karolchik D, Kent WJ (2013). Track data hubs enable visualization of user-defined genome-wide annotations on the UCSC Genome Browser.. Bioinformatics.

[R23] Rao Y, Zipursky SL (1998). Domain requirements for the Dock adapter protein in growth- cone signaling.. Proc Natl Acad Sci U S A.

[R24] Rao Y (2005). Dissecting Nck/Dock signaling pathways in Drosophila visual system.. Int J Biol Sci.

[R25] Rele CP, Sandlin KM, Leung W, Reed LK (2023). Manual annotation of Drosophila genes: a Genomics Education Partnership protocol.. F1000Res.

[R26] Schmucker D, Clemens JC, Shu H, Worby CA, Xiao J, Muda M, Dixon JE, Zipursky SL (2000). Drosophila Dscam is an axon guidance receptor exhibiting extraordinary molecular diversity.. Cell.

[R27] Song J, Wu L, Chen Z, Kohanski RA, Pick L (2003). Axons guided by insulin receptor in Drosophila visual system.. Science.

[R28] Stark K, Crowe O, Lewellyn L (2021). Precise levels of the Drosophila adaptor protein Dreadlocks maintain the size and stability of germline ring canals.. J Cell Sci.

[R29] Stevens A, Jacobs JR (2002). Integrins regulate responsiveness to slit repellent signals.. J Neurosci.

[R30] Tello-Ruiz MK, Marco CF, Hsu FM, Khangura RS, Qiao P, Sapkota S, Stitzer MC, Wasikowski R, Wu H, Zhan J, Chougule K, Barone LC, Ghiban C, Muna D, Olson AC, Wang L, Ware D, Micklos DA (2019). Double triage to identify poorly annotated genes in maize: The missing link in community curation.. PLoS One.

[R31] Weng YL, Liu N, DiAntonio A, Broihier HT (2011). The cytoplasmic adaptor protein Caskin mediates Lar signal transduction during Drosophila motor axon guidance.. J Neurosci.

[R32] Willoughby LF, Manent J, Allan K, Lee H, Portela M, Wiede F, Warr C, Meng TC, Tiganis T, Richardson HE (2017). Differential regulation of protein tyrosine kinase signalling by Dock and the PTP61F variants.. FEBS J.

[R33] Wu CL, Buszard B, Teng CH, Chen WL, Warr CG, Tiganis T, Meng TC (2011). Dock/Nck facilitates PTP61F/PTP1B regulation of insulin signalling.. Biochem J.

